# Molecular Characterization and Expression of *SPP1*, *LAP3* and *LCORL* and Their Association with Growth Traits in Sheep

**DOI:** 10.3390/genes10080616

**Published:** 2019-08-14

**Authors:** Yongfu La, Xiaoxue Zhang, Fadi Li, Deyin Zhang, Chong Li, Futao Mo, Weimin Wang

**Affiliations:** 1 College of Animal Science and Technology, Gansu Agricultural University, Lanzhou 730070, China; 2 Engineering Laboratory of Sheep Breeding and Reproduction Biotechnology in Gansu Province, Minqin 733300, China; 3 The State Key Laboratory of Grassland Agro-ecosystems, College of Pastoral Agriculture Science and Technology, Lanzhou University, Lanzhou 730020, China

**Keywords:** body weight, osteopontin, ovine genes, genetic characteristics

## Abstract

The *SPP1*, *LAP3*, and *LCORL* are located on chromosome 6 of sheep and a domain of 36.15-38.56 Mb, which plays an essential role in tissue and embryonic growth. In this study, we cloned the complete coding sequences of *SPP1* and partial coding regions of *LAP3* and *LCORL* from Hu sheep (Gansu Province, China) and analyzed their genomic structures. The RT-qPCR showed that the three genes were expressed widely in the different tissues of Hu sheep. The *SPP1* expression was significantly higher in the kidney (*p* < 0.01) and *LAP3* expression was significantly higher in the spleen, lung, kidney, and duodenum than in the other tissues (heart, liver, rumen, muscle, fat, and ovary; *p* < 0.05). The *LCORL* was preferentially expressed in the spleen, duodenum, and lung (*p* < 0.05). In addition, the nucleotide substitution NM_001009224.1:c.132A>C was found in *SPP1*; an association analysis showed that it was associated with birth weight and yearling weight (*p* < 0.05), and NM_001009224.1:c.132C was the dominant allele. Two mutations XM_012179698.3:c.232C>G and XM_012179698.3:c.1154C>T were identified in *LAP3*. The nucleotide substitution XM_012179698.3:c.232C>G was confirmed to be associated with birth weight, 1-month weight, 3-month weight (*p* < 0.05), and 2-month weight (*p* < 0.01). The nucleotide substitution XM_012179698.3:c.1154C>T was associated with birth weight (*p* < 0.01), 1-month weight, and 2-month weight (*p* < 0.05). The LAP3 gene XM_012179698.3:c.232C>G mutation with the C allele has higher body weight than other sheep, and CC genotype individuals show higher birth weight, 1-month weight, and weaning weight than the GG genotype individuals (*p* < 0.05). Our results support the conclusion that the mutations on ovine *SPP1* and *LAP3* successfully track functional alleles that affect growth in sheep, and these genes could be used as candidate genes for improving the growth traits of sheep during breeding.

## 1. Introduction

In sheep (*Ovis aries*), a region between 36.15 and 38.56 Mb on chromosome 6 (OAR6) includes 13 significant single nucleotide polymorphisms (SNPs) associated with body weight (BW) [1]. Secreted phosphoprotein 1 (SPP1), also known as osteopontin, is encoded by *SPP1*, and it was first identified as a major sialoprotein in the bone, helping osteoclasts to bind to the mineralized bone matrix [2]. The SPP1 is a multifunctional secreted glycosylated sialic-acid-rich phosphoprotein and an immobilized noncollagenous extracellular matrix protein in mineralized tissues [3]. In sheep, *SPP1* is located on OAR6, and the coding region on the cDNA is 837 bp. The *SPP1* is essential for vital biological processes, such as developmental processes, wound healing, immunological responses, tumorigenesis, bone resorption, and calcification [2]. Schnabel et al. [4] proposed that an SNP upstream of *SPP1* is a positional candidate polymorphism that explains a quantitative trait locus (QTL) on OAR6 that affects milk traits. White et al. [5] were able to detect significant effects of *SPP1* on the post-weaning growth of two populations of beef cattle. In addition, the association of *SPP1* with tissue growth [6] and embryonic growth [7] has been reported.

Leucine aminopeptidases (LAPs) are exopeptidases that catalyze the removal of N-terminal amino acids, and they belong to a family of aminopeptidases that have been found in many tissues, such as those of the kidney, pancreas, muscle, and liver and in mammary and subcellular locations in a variety of species [8]. The LAPs are often viewed as cell maintenance enzymes with critical roles in the turnover of peptides. The LAPs play important roles in cell maintenance, growth development, and defense [9], and they mainly participate in the organization updated the degradation of protein and peptide [10]. In sheep, *LAP3* is located on OAR6. Olsen et al. [11] mapped a QTL to a 420 kb region in bovine chromosome 6 that contains six milk production candidate genes, including *LAP3*. The *LAP3* encodes leucine aminopeptidase, which is associated with milk production traits, fat yield, and protein concentration in cattle [12]. However, there is little information on the *LAP3* in sheep.

Ligand dependent nuclear receptor corepressor like (LCORL) is located in the nucleus and closely associated with spermatogenesis [13]. Interestingly, *LCORL* has been consistently associated with human stature in genome-wide association studies [14] as well as with body size in dogs [15], cattle [16], and horses [17]. A previous study has shown that *LCORL* is associated with BW in Australian Merino sheep [1]. Rubin et al. [18] found that *LCORL* controls size variation in pigs.

There is much debate among researchers about the region between 36.15 and 38.56 Mb on OAR6. Moreover, little is known about the three genes in Hu sheep, and to the best of our knowledge, there is no information on the relationship between the three genes and BW traits of Hu sheep. Therefore, in this study, we cloned and molecularly characterized the complete or partial cDNA sequences of ovine *SPP1*, *LAP3*, and *LCORL* and analyzed their expression profiles in different tissues of Hu sheep. We also analyzed the association of the three genes with body weight of sheep.

## 2. Materials and Methods

### 2.1. Animals

All experiments in this study were carried out in accordance with the approved guidelines of the Regulation of the Standing Committee of Gansu People’s Congress. All experimental protocols and the collection of samples were approved by the Ethics Committee of Gansu Agriculture University under permission no. DK-005.

The sheep were obtained from a commercial sheep farm (Jinchang Zhongtian Sheep Industry Co. Ltd., Gansu, China) and allocated into two experimental groups: 204 Hu sheep (110 rams and 94 ewes) and 85 Hu sheep × (Dorper × Hu sheep) (35 rams and 50 ewes). The birth weight, 1-month weight, 2-month weight, 3-month weight, 4-month weight, 6-month weight, 8-month weight, 10-month weight, and 12-month weight were recorded. All efforts were made to minimize discomfort during blood collection. Blood samples for extraction of DNA were collected from the jugular vein under the supervision of qualified veterinarians. Venous jugular blood samples (5 mL) were obtained from each sheep and genomic DNA was extracted using the phenol-chloroform method. DNA was then dissolved in TE buffer (10 mM Tris-HCl, 1 mM EDTA, pH 8.0), and stored at −20 °C. Three female Hu sheep, each aged 35-days-old, were purchased from purebred herds from the same farm in Gansu province. The three selected sheep were healthy, similar in weight, and fed in an indoor setting under similar conditions of room temperature, illumination, feeding system, and nutrition level. The three female sheep were slaughtered, and tissues from the heart, liver, spleen, lung, kidney, rumen, duodenum, muscle, fat, hypothalamus, and hypophysis, were collected and immediately frozen in liquid nitrogen, then stored at −80 °C for RNA extraction.

### 2.2. cDNA Cloning and Sequence Analysis

The cDNA sequences of sheep *SPP1*, *LAP3*, and *LCORL* (GenBank Accession nos. NM_001009224.1, XM_012179698.3, XM_027970888.1, respectively) were used as templates. The primer pairs were designed using the coding regions of the three genes (Table 1). DNAstar software package (Madison, WI, USA) was used to predict the open reading frames (ORFs) of the cDNA sequences and calculate the amino acid sequences. The functions of the gene products were analyzed using Protfun software package (CBS, Lyngby, Denmark).

Total RNA was extracted from the tissues of an adult indigenous Hu sheep (Gansu Province, China) by using TransZol (TransGen Biotech, Beijing, China). The RT-PCR (reverse transcription polymerase chain reaction) was performed using Taq polymerase (TransGen Biotech, Beijing, China) and TransScript One-Step gDNA Removal and cDNA Synthesis SuperMix (TransGen Biotech, Beijing, China). The PCR product was purified using agarose gel DNA extraction kit (Takara, Dalian, China), and cloned into pMD18-T vector (volume of 10 μL of 50 ng DNA, 50 ng pMD18-T vector, 5 μL Solution I, incubated at 4 °C overnight). The recombinant DNA was transformed into DH5α competent cell and grown in LB (Luria-Bertani) agar plate with Amp, white colonies were selected (10 colonies for each sample) and cultured in liquid medium for 5 h, then submitted to the Shanghai Sangon Biological Engineering Company for sequencing. The sheep *SPP1*, *LAP3*, and *LCORL* gene cDNA sequences were compared with the sequenced sequences by BLAST analyses.

### 2.3. Tissue Expression Analysis of Sheep SPP1, LAP3, and LCORL

The mRNA levels of *SPP1*, *LAP3*, and *LCORL* were detected in tissues from the heart, liver, spleen, lung, kidney, rumen, duodenum, muscle, fat, hypothalamus, and hypophysis, of three Hu sheep. The total RNA from each tissue was extracted and reverse-transcribed into cDNA. Specific primers (*SPP1*-expression-S and *SPP1*-expression-A for *SPP1*, *LAP3*-expression-S, and *LAP3*-expression-A for *LAP3*, and *LCORL*-expression-S and *LCORL*-expression-A for *LCORL*; Table 1) for sheep *SPP1*, *LAP3*, and *LCORL* were used to amplify the products of 135, 170, and 280 bp, respectively. The PCR was performed at 94 °C for 5 min, followed by 34 cycles of 94 °C for 30 s, 57 °C for 30 s, and 72 °C for 30 s and a final extension at 72 °C for 5 min. *GAPDH* was used as the internal control gene. The qPCR was performed using the LightCycler 480II (Roche, Basel, Switzerland) and SYBR Green Realtime PCR Master Mix (Toyobo, Osaka, Japan). The 2-ΔΔCT method was used to analyze the data [19].

### 2.4. SNP Identification

The mutations of sheep *SPP1*, *LAP3* and *LCORL* were identified by sequencing the PCR products which were amplified using the eight DNA mixed samples of Hu sheep and Hu sheep × (Dorper × Hu sheep). The specific primers were designed on the basis of the assembled DNA sequences of the three genes of the sheep (Table 1). Additionally, the primers were used for PCR-restriction fragment length polymorphism (PCR-RFLP). The DNA was extracted from the blood of 289 Hu sheep (*n* = 204; 110 rams and 94 ewes) and Hu sheep × (Dorper × Hu sheep) (*n* = 85; 35 rams and 50 ewes). The PCR for genotyping was performed using a reaction volume of 25 μL that consisted of 1×EasyTaq^®^ PCR SuperMix (+dye) (TransGen Biotech, Beijing, China), 50 ng of genomic DNA, and 8 pmol of each primer, the rest of the volume was made up by ddH_2_O. The PCR parameters for *SPP1*, *LAP3*, and *LCORL* were 94 °C for 5 min, followed by 35 cycles of 94 °C for 30 s, 52–58 °C for 30–90 s, and 72 °C for 30 s and a final extension of 72 °C for 5 min. The 4 μL PCR product was digested for 60 min with 2 U of *SmlI* for *SPP1*, *AcuI* and *BccI* for *LAP3*, and *Hpy188I* and *DraI* for *LCORL* at 37 °C and then separated on a 3% agarose gel stained with GelRed.

### 2.5. Association Analysis

The PROC GLM procedure in SAS software package (SAS Institute Inc., Cary, NC, USA) was used to analyze the association between the genotypes and trait. The linear model with the fixed effects was as follows:

Y*_ijklm_* = *μ* + G*_i_* + B*_j_* + B*_k_* + S*_l_* + C*_m_* + ε*_ijklm_*

where Y*_ijklm_* is the *ijklmn*^th^ trait observation value; *μ* is the mean; G*_i_* is the effect of the *i*^th^ genotype; B*_j_* is the effect of the *j*^th^ farm; B*_k_* is the effect of the *j*^th^ breeding; S*_l_* is the effect of the *j*^th^ sex; C*_m_* is the effect of the combination; and ε*_ijklm_* is the residual corresponding to the trait observation value with var(ε) = Iσ^2^_e_. B*_j_*, B*_k_*, S*_l_*, and C*_m_* are the fixed effects. *p* < 0.05 was considered as the statistically significant criterion.

## 3. Results

### 3.1. Molecular Cloning and Sequence Analysis of Sheep SPP1, LAP3, and LCORL

In this study, 1122 bp of the sheep *SPP1* gene was cloned, which contained a calculated ORF of 837 bp encoding a protein of 278 amino acid residues. Additionally, sheep *LAP3* and *LCORL* contain ORFs of 1560 and 3117 bp, respectively, and they encode proteins of 519 and 1038 amino acid residues, respectively. The molecular weights of *SPP1*, *LAP3*, and *LCORL* are 31.1 kDa, 56.2 kDa, and 118.2 kDa, respectively, and the theoretical isoelectric points are 4.15, 6.44, and 10.36, respectively.

**Figure 1:**
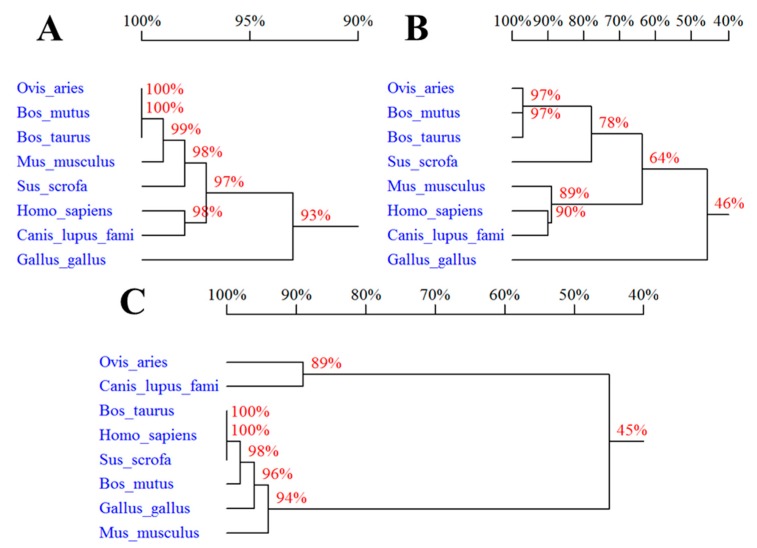
Percentage of sequences homology of *SPP1* (**A**), *LAP3* (**B**), and *LCORL* (**C**): *Bos taurus* (NP_776612.1, NP_776523.2, and NP_001179286.1), *Bos mutus* (XP_005897856.1, XP_005887081.1, and XP_005897159.1), *Homo sapiens* (NP_000573.1, NP_056991.2, and NP_001159611.1), *Sus scrofa* (NP_999188.1, XP_003356918.4, and NP_001182274.1), *Gallus gallus domesticus* (NP_989866.1, NP_001026507.1, and NP_001026331.1), *Canis lupus familiaris* (XP_003434072.1, XP_005618600.1, and XP_013967832.1), and *Mus musculus* (NP_001191130.1, NP_077754.3, and NP_001156545.1).

Percentage of sequences homology of the three proteins in *Ovis aries*, *Bos taurus*, *Bos mutus*, *Homo sapiens*, *Sus scrofa*, *Gallus gallus domesticus*, *Canis lupus familiaris*, and *Mus musculus* showed that *Ovis aries* SPP1, LAP3 and LCORL are most similar to *Bos mutus* SPP1 (99%), *Bos mutus* LAP3 (100%), and *Canis lupus familiaris* LCORL (87%), respectively (Figure 1).

### 3.2. Expression Profile Analysis

**Figure 2:**
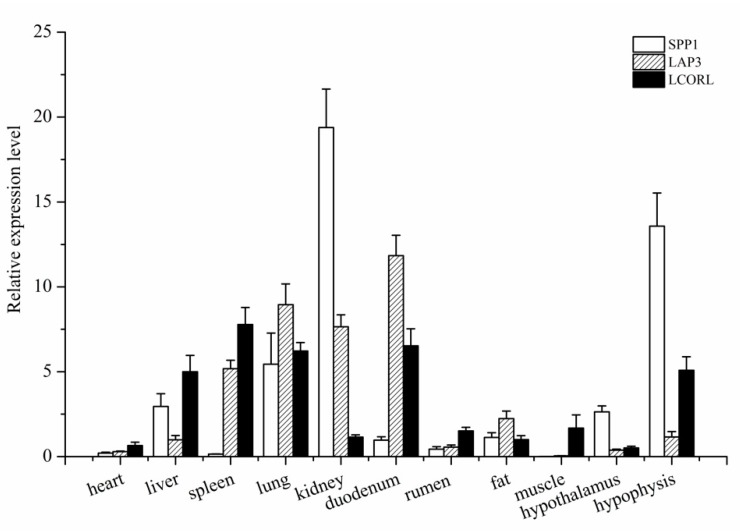
The mRNA expression profiles of *SPP1* (**A**), *LAP3* (**B**), and *LCORL* (**C**) in various tissues of sheep. The expressing of these three genes was by qPCR and normalized to the expression of *GAPDH*, each sample was amplified in triplicate and the data were shown as mean ± standard deviation.

The RT-qPCR was used to investigate the general tissue distributions of *SPP1*, *LAP3*, and *LCORL*, and the results showed that the three genes were widely expressed (Figure 2). They were detected in all eleven tissues, namely, heart, liver, spleen, lung, kidney, rumen, duodenum, muscle, fat, hypothalamus, and pituitary tissues. The *SPP1* was expressed in 11 tissues of Hu sheep, with the highest level in the kidney (*p* < 0.01), followed by the hypothalamus (*p* < 0.05). The *LAP3* expression was significantly higher in spleen, lung, kidney, and duodenum than in the other tissues (heart, liver, rumen, muscle, fat, and ovary; *p* < 0.05). The *LCORL* was preferentially expressed in spleen, duodenum, and lung (*p* < 0.05).

### 3.3. SNPs of Sheep SPP1, LAP3, and LCORL

**Figure 3:**
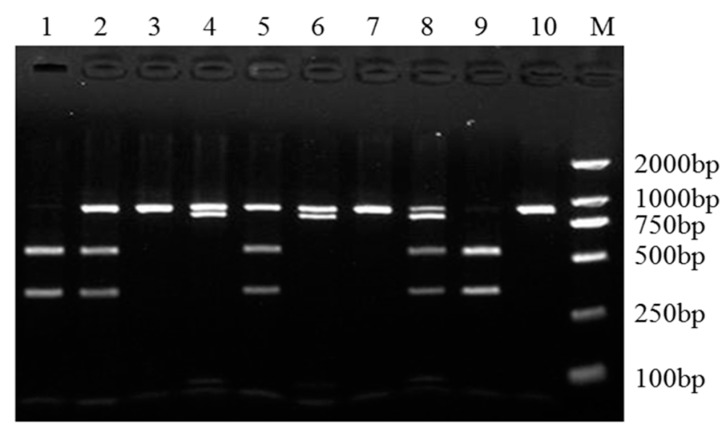
The PCR-restriction fragment length polymorphism (PCR-RFLP) results for the different genotypes of sheep *SPP1*. The PCR products digested with *SmlI* showed different genotypes of *SPP1* NM_001009224.1:c.132A>C and NC_040257.1(NM_001009224.1):c.174+402G>A mutations, of which NM_001009224.1:c.132A>C mutation has three genotypes (AA, AC and CC), and NC_040257.1(NM_001009224.1):c.174+402G>A mutation has two genotypes (GG and GA). Five combined genotypes were detected using *SmlI* in this experimental population, namely AAGG (lanes 3, 7, 10), AAGA (lanes 4, 6), ACGG (lanes 2, 5), ACGA (lane 8), and CCGG (lanes 1, 9). M: DNA Marker DL2000.

We recorded two nucleotide substitutions NM_001009224.1:c.132A>C and NC_040257.1(NM_001009224.1):c.174+402G>A in sheep *SPP1*. For two substitutions, the length of the amplified fragment was 885 bp, and the substitutions were locating at 359 and 803 bp, recognized by *SmlI* (Figure S1). When nucleotide A is substituted by G at NC_040257.1(NM_001009224.1):c.174+402, the nucleotide substitution NM_001009224.1:c.132A>C was detected using *SmlI*, which yielded three fragments: 885 bp band representing allele T, and 359 and 526 bp bands representing allele G. When nucleotide G is substituted by A at NC_040257.1(NM_001009224.1):c.174+402, the nucleotide substitution NM_001009224.1:c.132A>C was detected using *SmlI*, which yielded four fragments: 803 and 82 bp bands representing allele A, 359, and 444 and 82 bp bands representing allele C. When nucleotide C is substituted by A at NM_001009224.1:c.132, the nucleotide substitution NC_040257.1(NM_001009224.1):c.174+402G>A was detected using *SmlI*, which yielded three fragments: 885 bp band representing allele G, and 803 and 82 bp bands representing allele A. When nucleotide A is substituted by C at NM_001009224.1:c.132, the nucleotide substitution NC_040257.1(NM_001009224.1):c.174+402G>A was detected using *SmlI*, which yielded four fragments: 359 and 526 bp bands representing allele G, and 359, 444 and 82 bp bands representing allele A (Figure 3).

**Figure 4:**
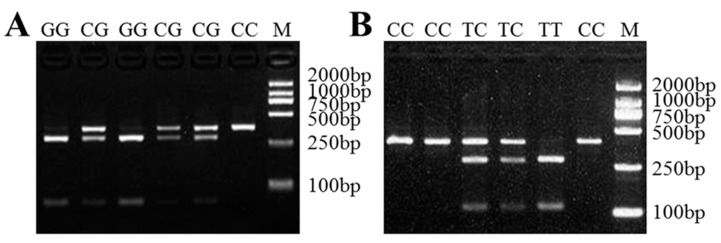
The PCR-RFLP results for the different genotypes of sheep *LAP3*. The genotypes are marked at the top of the lanes. (**A**) The PCR products digested using *AcuI* show the different genotypes of *LAP3* XM_012179698.3:c.232C>G mutation. (**B**) The PCR products digested using *BccI* show the different genotypes of *LAP3* XM_012179698.3:c.1154C>T mutation. M: DNA Marker DL2000.

We recorded two nucleotide substitutions XM_012179698.3:c.232C>G and XM_012179698.3:c.1154C>T in sheep *LAP3*. For the first substitution, the length of the amplified fragment was 351 bp, with the nucleotide substitution locating at 281 bp recognized by *AcuI* (Figure S2), which generated three fragments: 351 bp band representing allele C, and 281 and 70 bp bands representing allele G. The nucleotide substitution XM_012179698.3:c.1154C>T was detected using *BccI* (Figure S3), which yielded three fragments: 407 bp band representing allele C, and 292 and 115 bp bands representing allele T (Figure 4).

**Figure 5:**
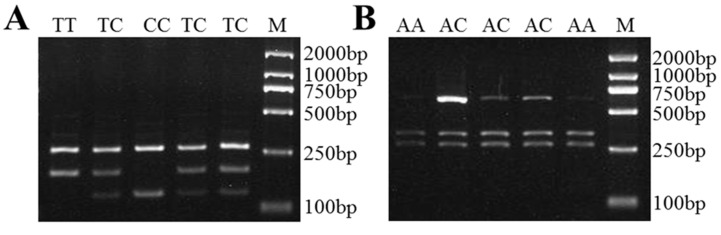
The PCR-RFLP results for the different genotypes of sheep *LCORL*. The genotypes are marked at the top of the lanes. (**A**) The PCR products digested using *Hpy188I* show the different genotypes of *LCORL* XM_027970888.1:c.-1096T>C mutation. (**B**) The PCR products digested using *DraI* show the different genotypes of *LCORL* XM_027970888.1:c.2162A>C mutation. M: DNA Marker DL2000.

We recorded two substitutions XM_027970888.1:c.-1096T>C and XM_027970888.1:c.2162A>C in sheep *LCORL*. For the first substitution, the length of the amplified fragment was 495 bp, with the nucleotide substitutions locating at 281 and 414 bp and fixed sequences TCAGA at 277-281 bp and TCGGA at 413-417 bp recognized by *Hpy188I*, which generated four fragments: 281 and 214 bp bands representing allele T, 281, 136 and 78 bp bands representing allele C (Figure S4). The nucleotide substitution XM_027970888.1:c.2162A>C was detected using *DraI* (Figure S5), which yielded three fragments: 322 and 272 bp bands representing allele A and 594 bp band representing allele C (Figure 5).

### 3.4. Association of Sheep SPP1, LAP3, and LCORL with BW

The effect of the sheep *SPP1* variation on BW of the experimental populations was studied. The results show that the NC_040257.1(NM_001009224.1):c.174+402G>A mutation of sheep *SPP1* has no association with BW. In contrast, the *SPP1* NM_001009224.1:c.132A>C substitution was associated with birth weight and 12-month weight (*p* < 0.05; Table 2). Moreover, all the phenotype values of birth weight and 12-month weight in the animals with AA and CC genotypes were evidently higher than those with the AC genotype (*p* < 0.05). This indicated that the homozygote contributed higher phenotype values than the heterozygote.

The effect of sheep *LAP3* nucleotide substitution on the body weight of the experimental populations was also studied. The results showed that the XM_012179698.3:c.232C>G substitution was associated with birth weight, 1-month weight, 3-month weight (*p* < 0.05), and 2-month weight (*p* < 0.01; Table 2). Besides, all the phenotype values of the animals with the CG genotype were evidently higher than those with the GG genotype, whereas the difference between CC and CG was not significant (*p* > 0.05). This indicated that allele C contributed higher phenotype values than allele G. The XM_012179698.3:c.1154C>T substitution had a significant effect on birth weight (*p* < 0.01), 1-month weight, and 2-month weight (*p* < 0.05; Table 2). All the phenotype values for BW of the animals with the CC genotype were evidently higher than those with the TT genotype. This indicated that allele C contributed higher phenotype values than allele T. In addition, the association analysis showed no correlation between the two substitutions of *LCORL* and body weight (Table 2).

## 4. Discussion

In this study, the multiple amino acid sequence alignments show that SPP1 is more conserved than LAP3 and LCORL across the above-mentioned eight species. The SPP1 is a highly phosphorylated protein containing a polyaspartic acid sequence and a conserved RGD motif, and plays important roles in physiological processes such as inflammatory responses, calcification, organ development, immune cell function and carcinogenesis [20]. In vitro, *SPP1* is a potent, partial agonist of cortical and hippocampal M1 receptors with activity conserved across species [21]. The region between −112 and −62 bp of the *SPP1* promoter is highly conserved in the rat, mouse and human promoters and contains a number of consensus regions, including an E-box and a GC-rich region [22]. Hijiya et al. isolated the human *SPP1* and the 5′ upstream region, and analyzed its exon–intron structure and potential regulatory sequences of the promoter region in comparison with those of the mouse and porcine gene. They found that the 5′ upstream region of the *SPP1*, which is highly conserved up to nucleotide −250, contains a number of potential cis regulatory consensus sequences [23]. Results of all these previous studies indicate that the *SPP1* is highly conserved between different species. At present, there are few studies on the *LAP3* and *LCORL*. In this study, we analyzed the homology of sheep SPP1, LAP3, and LCOR proteins with seven other species, respectively. It was found that SPP1 has a higher percentage of sequences homology indicating that SPP1 is more conserved than LAP3 and LCORL across the above-mentioned eight species. The tissue expression profiles revealed that *SPP1* has a broad expression pattern in Hu sheep.

The SPP1 is a multifunctional glycosylated phosphoprotein that participates in many physiological and pathological processes, and it is expressed in multiple tissues and organs, such as the kidney and liver, and the central nervous system [3]. The LAP3 catalyzes the removal of N-terminal amino acid, and it belongs to a family of aminopeptidases involved in protein maturation and degradation and found in many tissues [24,25]. Thus, our results were generally consistent with those of previous studies. In addition, *LCORL* is a transcription factor that may function during spermatogenesis in the testes [13]. The RT-PCR results showed that *LCORL* was widely expressed and detected in all eleven tissues: heart, liver, spleen, lung, kidney, rumen, duodenum, muscle, fat, hypothalamus, and hypophysis. The high mRNA levels in the spleen, lung, liver, and duodenum could be attributed to their crucial roles as immune and uptake organs.

We identified two mutations, NM_001009224.1:c.132A>C and NC_040257.1(NM_001009224.1):c.174+402G>A, in sheep *SPP1*. The association analysis of sheep *SPP1* showed that the novel nucleotide substitution NM_001009224.1:c.132A>C had significant effects (*p* < 0.05) on birth weight and yearling weight, whereas no correlation was detected for NC_040257.1(NM_001009224.1):c.174+402G>A. Previous studies have reported that *SPP1* has significant effects on the birth weight and weaning weight of beef cattle [5] and BW of Australian Merino sheep [26]. The association analysis revealed that the XM_012179698.3:c.232C>G mutation of *LAP3* was associated with the birth weight (*p* < 0.05), 1-month weight (*p* < 0.05), 2-month weight (*p* < 0.01), and 3-month weight (*p* < 0.05). Moreover, a significant association was observed between the XM_012179698.3:c.1154C>T mutation of *LAP3* and birth weight (*p* < 0.01), 1-month weight (*p* < 0.05), and 2-month weight (*p* < 0.05), and allele C was the preponderant allele. Allan et al. [26] found that *LAP3* was significantly associated with the body weight of Australian Merino sheep, which is consistent with our results using Hu sheep and their crossed offspring. The *LCORL* has been associated with the average daily gain of cattle [13]. However, the two mutations detected in sheep *LCORL* had no association in Hu sheep and its filial generation, which may be due to the differences between varieties. Thus, *SPP1* and *LAP3* can be used as molecular markers for improving the growth performance of sheep.

Our results indicate that *SPP1* and *LAP3* can be used as candidate genes for improving the body weight of sheep during breeding. However, further studies on the association between the three genes and growth performance of different sheep breeds are required.

## Figures and Tables

**Table 1:** Primer pairs designed for sheep genes secreted phosphoprotein 1 (*SPP1*), leucine aminopeptidase 3 (*LAP3*), and ligand dependent nuclear receptor corepressor like (*LCORL*).

Primer Name	Primer Sequence (5′-3′)	Annealing Temperature (°C)	Size (bp)
*SPP1*-CDS-S	CATCAGCATCACAGGGGACT	57	1122
*SPP1*-CDS-A	GGAAAGAACATAGACTAAACCCT		
*SPP1*-expression-S	ATGACTCCGACGATGCTGAAC	57	135
*SPP1*-expression-A	CGTAGGGAAAGGTGGAGTG		
*SPP1*-SNP-S	GGACAGAGGCTGAAGGAATAC	58	885
*SPP1*-SNP-A	CATCCAAAGCAGGTCTTAT		
*LAP3*-CDS-S	TCGGTGGAGGGCGGTACG	55	567
*LAP3*-CDS-A	GAAGATAAGGAACCTCAT		
*LAP3*-expression-S	TGCCCATCAACATTGTAGGT	60	170
*LAP3*-expression-A	AGTGTGAGCGTAGCAGAGCG		
*LAP3*-SNP_1_-S	GGCACTGCTTTCTATCATTG	55	351
*LAP3*- SNP_1_-A	ATAGGTGTTCACTGAGGGTT		
*LAP3*-SNP_2_-S	CTTTTAGTCTTTTGACCTTC	55	407
*LAP3*-SNP_2_–A	GCTTTGTATCATTTTTAGCT		
*LCORL*-CDS-S	AACTGACCAAACCGACAT	54	1543
*LCORL*-CDS-A	TATCCAAGCACCTGTCCC		
*LCORL*-expression-S	CTGCTTACCTCCTTTAGA	52	280
*LCORL*-expression-A	GTCCTCCTGACTTTTACC		
*LCORL*–SNP_1_-S	AGAGTCTCAGAATCCCCTAA	52	495
*LCORL*–SNP_1_-A	TTGCTTATTTCTGCTGGTGT		
*LCORL*-SNP_2_-S	GAACCCATTGAAAACGATAA	55	594
*LCORL*-SNP_2_-A	AGGTGGGAAAATAAACTGAT		
*GAPDH*-expression-S	GGGGTCTACACTCCCAACTGC	58	379
*GAPDH*-expression-A	CAGAAGGCGGCGATGGAA		

**Table 2:** Associations between the genotypes of the single nucleotide polymorphisms (SNPs) and body weight in the experimental populations.

Gene Name	Locus	Genotype	n	Live Weight								
				Birth	1-month	2-month	3-month	4-month	6-month	8-month	10-month	12-month
*SPP1*	NM_001009224.1:c.132A>C	AA	143	3.499 ± 0.061^a^	11.227 ± 0.275	18.041 ± 0.351	23.231 ± 0.387	28.135 ± 0.419	33.656 ± 0.472	35.036 ± 0.494	41.245 ± 0.769	46.384 ± 0.829^a^
		AC	102	3.354 ± 0.074^b^	11.082 ± 0.331	17.825 ± 0.423	22.999 ± 0.466	28.223 ± 0.506	34.610 ± 0.569	35.408 ± 0.595	40.345 ± 0.927	44.818 ± 1.000^b^
		CC	44	3.569 ± 0.103^a^	11.507 ± 0.462	17.791 ± 0.591	23.300 ± 0.651	27.800 ± 0.706	33.422 ± 0.794	34.817 ± 0.831	41.667 ± 1.294	47.383 ± 1.396^a^
*LAP3*	XM_012179698.3: c.232C>G	CC	104	3.449 ± 0.067^a^	11.257 ± 0.295^a^	18.029 ± 0.372^A^	23.275 ± 0.415^a^	28.075 ± 0.451	33.792 ± 0.511	35.144 ± 0.533	41.252 ± 0.830	46.067 ± 0.901
		CG	139	3.500 ± 0.074^a^	11.251 ± 0.327^a^	18.034 ± 0.412^A^	23.393 ± 0.459^a^	28.429 ± 0.499	34.163 ± 0.566	35.019 ± 0.590	40.791 ± 0.919	46.124 ± 0.998
		GG	46	3.285 ± 0.099^b^	10.865 ± 0.441^b^	17.535 ± 0.556^B^	22.799 ± 0.619^b^	28.466 ± 0.673	34.778 ± 0.763	36.051 ± 0.795	41.114 ± 1.240	45.764 ± 1.345
	XM_012179698.3: c.1154C>T	CC	86	3.334 ± 0.072^Aa^	10.195 ± 0.338^a^	16.457 ± 0.434^a^	21.671 ± 0.460	26.294 ± 0.526	31.857 ± 0.601	33.359 ± 0.609	38.990 ± 1.001	43.528 ± 1.082
		TC	129	3.268 ± 0.054^ABa^	9.652 ± 0.252^ab^	15.862 ± 0.323^ab^	21.510 ± 0.343	25.795 ± 0.392	31.432 ± 0.448	33.007 ± 0.454	38.021 ± 0.747	42.794 ± 0.807
		TT	74	3.080 ± 0.071^Bb^	9.135 ± 0.332^b^	15.088 ± 0.426^b^	21.097 ± 0.452	25.850 ± 0.516	31.819 ± 0.590	33.042 ± 0.598	38.489 ± 0.983	43.516 ± 1.062
*LCORL*	XM_027970888.1: c.-1096T>C	CC	132	3.241 ± 0.056	9.414 ± 0.261	15.496 ± 0.338	21.131 ± 0.357	25.478 ± 0.408	31.569 ± 0.458	33.278 ± 0.460	38.874 ± 0.765	43.804 ± 0.841
		TC	118	3.214 ± 0.054	9.740 ± 0.253	15.896 ± 0.328	21.495 ± 0.346	26.090 ± 0.396	31.507 ± 0.444	32.873 ± 0.446	37.835 ± 0.741	42.529 ± 0.815
		TT	39	3.269 ± 0.117	10.145 ± 0.546	16.798 ± 0.708	22.138 ± 0.748	26.663 ± 0.885	31.811 ± 0.959	32.512 ± 0.963	37.827 ± 1.602	43.112 ± 1.760
	XM_027970888.1: c.2162A>C	AA	225	3.219 ± 0.040	9.529 ± 0.187	15.930 ± 0.244	21.424 ± 0.255	26.010 ± 0.291	31.872 ± 0.327	33.322 ± 0.372	38.401 ± 0.556	43.261 ± 0.608
		AC	64	3.241 ± 0.072	9.732 ± 0.337	15.561 ± 0.441	21.198 ± 0.462	25.247 ± 0.526	30.770 ± 0.592	32.228 ± 0.592	37.121 ± 1.005	41.911 ± 1.098

Note: Different capital-case letters in the same group indicate highly significant difference (*p* < 0.01), and different lower-case letters in the same group indicate significant difference (*p* < 0.05).

## Supplementary Materials

The following are available online at https://www.mdpi.com/2073-4425/10/8/616/s1, Figure S1: The SPP1 gene SNPs and restriction recognition sites of sheep. The underlined for primers, the red font for restricted recognition sites, Figure S2: The LAP3 gene XM_012179698.3:c.232C>G substitution and its restriction recognition site of sheep. The underlined for primers, the red font for restricted recognition site, Figure S3: The LAP3 gene XM_012179698.3:c.1154C>T substitution and its restriction recognition site of sheep. The underlined for primers, the red font for restricted recognition site, Figure S4: The LCORL gene XM_027970888.1:c.-1096T>C substitution and its restriction recognition site of sheep. The underlined for primers, the red font for restricted recognition site, Figure S5: The LCORL gene XM_027970888.1:c.2162A>C substitution and its restriction recognition site of sheep. The underlined for primers, the red font for restricted recognition site.
