# Supplementary material for: Molecular Characterization and Expression of SPP1, LAP3 and LCORL and Their Association with Growth Traits in Sheep
Source: Genes (Basel). 2019 Aug 14;10(8):616. doi: 10.3390/genes10080616 (PMC6723280; doi:10.3390/genes10080616)
Supplement: Supplementary file 1 [file genes-10-00616-s001.zip › Supplementary fileú¿annotation for supplementary tables ).docx]

**Additional files:**

Figure S1. The SPP1 gene SNPs and restriction recognition sites of sheep. The underlined for primers, the red font for restricted recognition sites.

Figure S2. The LAP3 gene XM_012179698.3:c.232C>G substitution and its restriction recognition site of sheep. The underlined for primers, the red font for restricted recognition site.

Figure S3. The LAP3 gene XM_012179698.3:c.1154C>T substitution and its restriction recognition site of sheep. The underlined for primers, the red font for restricted recognition site.

Figure S4. The LCORL gene XM_027970888.1:c.-1096T>C substitution and its restriction recognition site of sheep. The underlined for primers, the red font for restricted recognition site.

Figure S5. The LCORL gene XM_027970888.1:c.2162A>C substitution and its restriction recognition site of sheep. The underlined for primers, the red font for restricted recognition site.
