# Supplementary figures and images for: Molecular Characterization and Expression of SPP1, LAP3 and LCORL and Their Association with Growth Traits in Sheep
Source: Genes (Basel). 2019 Aug 14;10(8):616. doi: 10.3390/genes10080616 (PMC6723280; doi:10.3390/genes10080616)

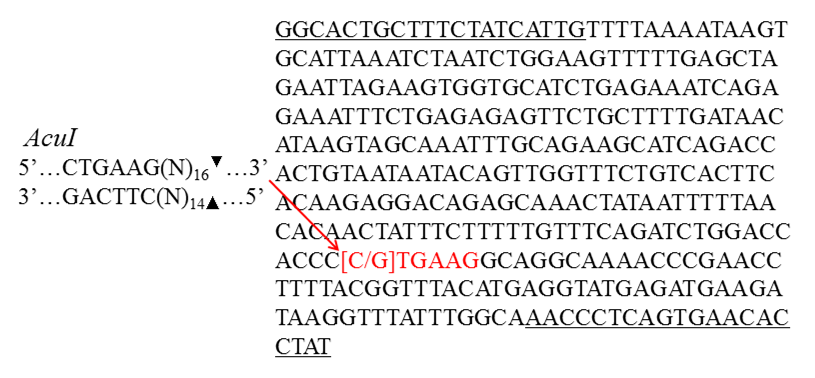

Supplement: Supplementary file 1 [file genes-10-00616-s001.zip › Figure S2.png]

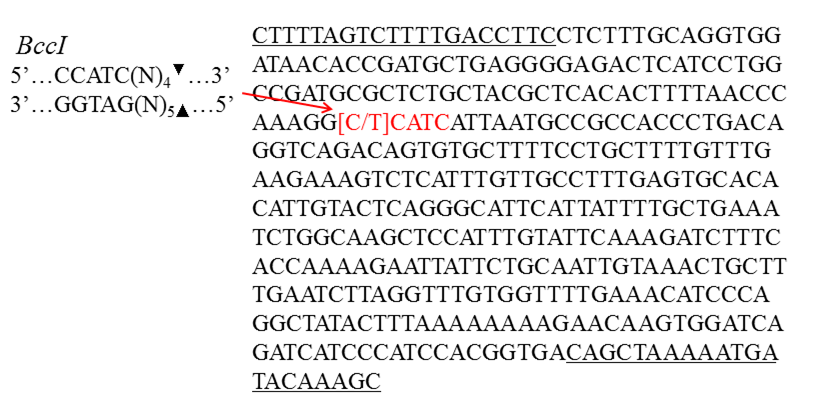

Supplement: Supplementary file 1 [file genes-10-00616-s001.zip › Figure S3.png]

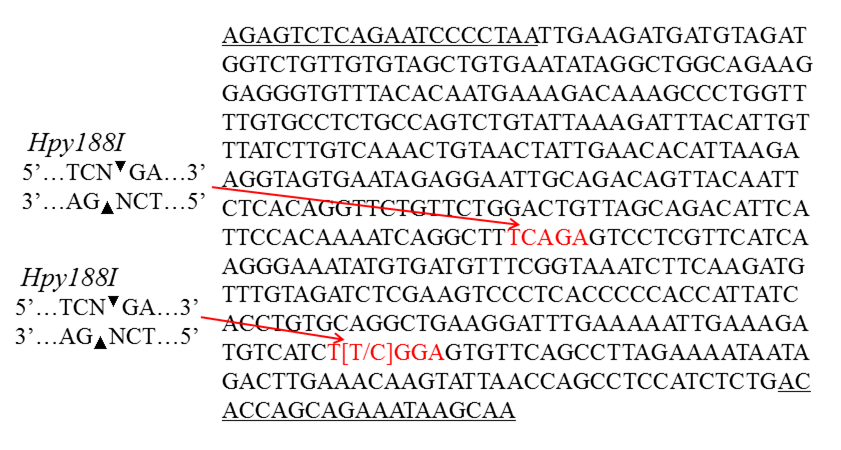

Supplement: Supplementary file 1 [file genes-10-00616-s001.zip › Figure S4.png]

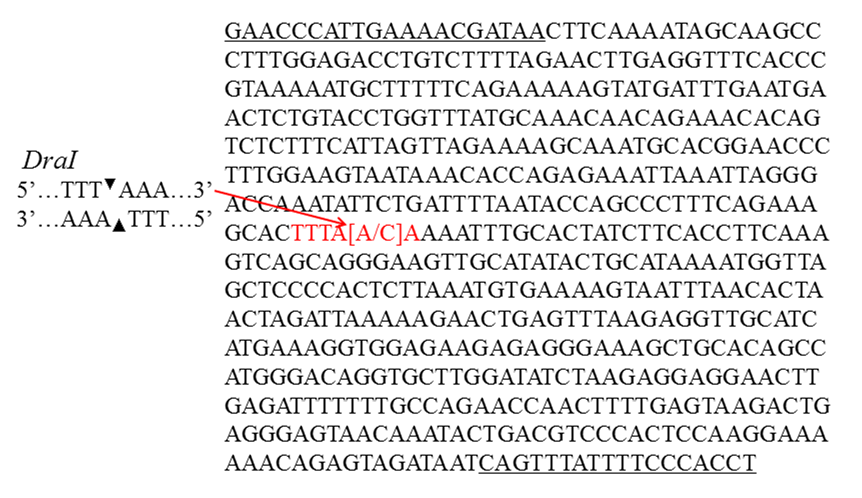

Supplement: Supplementary file 1 [file genes-10-00616-s001.zip › Figure S5.png]

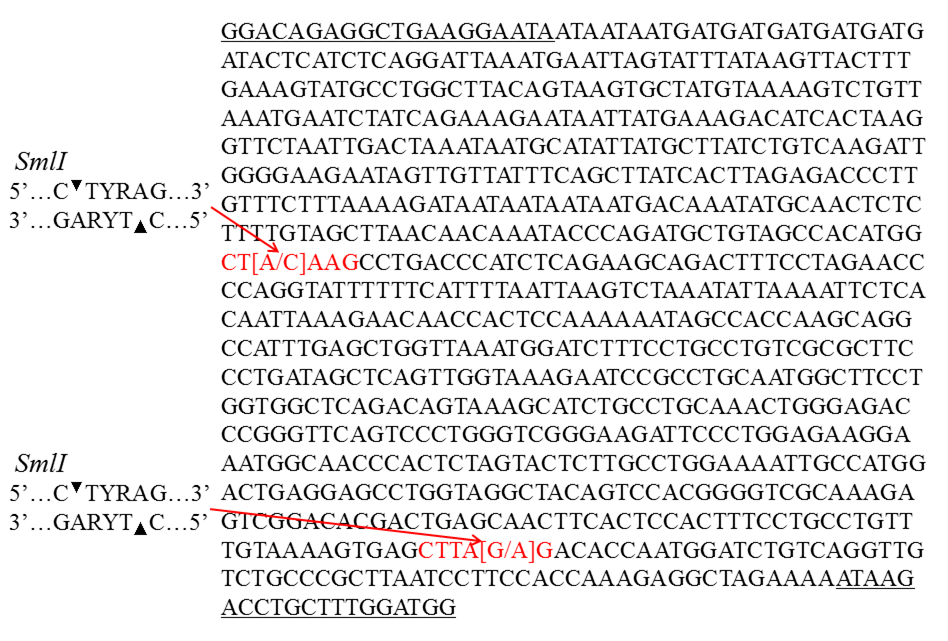

Supplement: Supplementary file 1 [file genes-10-00616-s001.zip › Figure S1.png]
